# Multi-Disease Segmentation of Gliomas and White Matter Hyperintensities in the BraTS Data Using a 3D Convolutional Neural Network

**DOI:** 10.3389/fncom.2019.00084

**Published:** 2019-12-20

**Authors:** Jeffrey D. Rudie, David A. Weiss, Rachit Saluja, Andreas M. Rauschecker, Jiancong Wang, Leo Sugrue, Spyridon Bakas, John B. Colby

**Affiliations:** ^1^Department of Radiology, Perelman School of Medicine, University of Pennsylvania, Philadelphia, PA, United States; ^2^Department of Radiology & Biomedical Imaging, University of California, San Francisco, San Francisco, CA, United States; ^3^Department of Bioengineering, University of Pennsylvania, Philadelphia, PA, United States; ^4^Department of Electrical and Systems Engineering, University of Pennsylvania, Philadelphia, PA, United States; ^5^Center for Biomedical Image Computing and Analytics (CBICA), University of Pennsylvania, Philadelphia, PA, United States; ^6^Department of Pathology and Laboratory Medicine, Perelman School of Medicine, University of Pennsylvania, Philadelphia, PA, United States

**Keywords:** segmentation, glioblastoma, convolutional neural network, white matter hyperintensities, deep learning, radiology, multi-disease classification

## Abstract

An important challenge in segmenting real-world biomedical imaging data is the presence of multiple disease processes within individual subjects. Most adults above age 60 exhibit a variable degree of small vessel ischemic disease, as well as chronic infarcts, which will manifest as white matter hyperintensities (WMH) on brain MRIs. Subjects diagnosed with gliomas will also typically exhibit some degree of abnormal T2 signal due to WMH, rather than just due to tumor. We sought to develop a fully automated algorithm to distinguish and quantify these distinct disease processes within individual subjects’ brain MRIs. To address this multi-disease problem, we trained a 3D U-Net to distinguish between abnormal signal arising from tumors vs. WMH in the 3D multi-parametric MRI (mpMRI, i.e., native T1-weighted, T1-post-contrast, T2, T2-FLAIR) scans of the International Brain Tumor Segmentation (BraTS) 2018 dataset (*n*_training_ = 285, *n*_validation_ = 66). Our trained neuroradiologist manually annotated WMH on the BraTS training subjects, finding that 69% of subjects had WMH. Our 3D U-Net model had a 4-channel 3D input patch (80 × 80 × 80) from mpMRI, four encoding and decoding layers, and an output of either four [background, active tumor (AT), necrotic core (NCR), peritumoral edematous/infiltrated tissue (ED)] or five classes (adding WMH as the fifth class). For both the four- and five-class output models, the median *Dice* for whole tumor (WT) extent (i.e., union of AT, ED, NCR) was 0.92 in both training and validation sets. Notably, the five-class model achieved significantly (*p* = 0.002) lower/better Hausdorff distances for WT extent in the training subjects. There was strong positive correlation between manually segmented and predicted volumes for WT (*r* = 0.96) and WMH (*r* = 0.89). Larger lesion volumes were positively correlated with higher/better *Dice* scores for WT (*r* = 0.33), WMH (*r* = 0.34), and across all lesions (*r* = 0.89) on a log(10) transformed scale. While the median *Dice* for WMH was 0.42 across training subjects with WMH, the median *Dice* was 0.62 for those with at least 5 cm^3^ of WMH. We anticipate the development of computational algorithms that are able to model multiple diseases within a single subject will be a critical step toward translating and integrating artificial intelligence systems into the heterogeneous real-world clinical workflow.

## Introduction

A significant challenge in the deployment of advanced computational methods into typical clinical workflows is the vast heterogeneity of disease processes, which are present both between individuals (inter-subject heterogeneity) and within individuals (intra-subject heterogeneity). Most adults over the age of 60 have a variable degree of abnormal signal on brain MRIs due to age-related changes manifesting as white matter hyperintensities (WMH), which are typically secondary to small vessel ischemic disease (SVID) and chronic infarcts that can be found in subjects with vascular risk factors and clinical histories of stroke and dementia (Wardlaw et al., 2015). These lesions can confound automated detection and segmentation of other disease processes, including brain tumors, which also result in abnormal signal in T2-weighted (T2) and T2 Fluid-attenuated inversion recovery (T2-FLAIR) MRI scans secondary to neoplastic processes and associated edema/inflammation. We sought to address this challenge of intra-individual heterogeneity by leveraging (i) the dataset of the International Multimodal Brain Tumor Segmentation (BraTS) 2018 challenge (Menze et al., 2015; Bakas et al., 2017b, 2019) (ii) expert radiologist expertise, and (iii) three-dimensional (3D) convolutional neural networks (CNNs).

Advances in the field of segmentation and radiomics within neuro-oncology have been supported by data made available through The Cancer Imaging Archive (TCIA; Clark et al., 2013). Since 2012, the BraTS challenge has further curated TCIA glioma multi-parametric MRI (mpMRI) scans, segmentation of tumor sub-regions, and survival data in a public dataset and sponsored a yearly challenge to improve performance of automated segmentation and prognostication methods (Menze et al., 2015; Bakas et al., 2017b, 2019). Similar to BraTS, there have been large efforts for improving automatic segmentation of WMH (Griffanti et al., 2016; Habes et al., 2016), which include the MICCAI 2017 WMH competition (Li et al., 2018; Kuijf et al., 2019), as well as stroke lesions, through the Ischemic Stroke Lesion Segmentation Challenge (ISLES; Winzeck et al., 2018). Deep learning (DL) approaches for biomedical image segmentation are now established as superior to the previous generation of atlas-based and hand-engineered feature approaches (Fletcher-Heath et al., 2001; Gooya et al., 2012), as demonstrated by their performance in recent image segmentation challenges (Chang, 2017; Kamnitsas et al., 2017; Li et al., 2018; Bakas et al., 2019; Myronenko, 2019).

Deep learning relies on hierarchically organized layers to process increasingly complex intermediate feature maps and utilizes the gradient of the error in predictions with regard to the units of each layer to update model weights, known as “back-propagation.” In visual tasks, this allows for the identification of lower- and intermediate-level image information (feature maps) to maximize classification performance based on annotated datasets (LeCun et al., 2015; Chartrand et al., 2017; Hassabis et al., 2017). Typically, CNNs, a class of feed-forward neural networks, have been used for image-based problems, achieving super-human performance in the ImageNet challenge (Deng et al., 2009. Krizhevsky et al., 2012). The U-Net architecture (Ronneberger et al., 2015; Cicek et al., 2016; Milletari et al., 2016) describes a CNN with an encoding convolutional arm and corresponding decoding [de]convolutional arm has been shown to be particularly useful for 3D biomedical image segmentation through its semantic- and voxel-wise approach, such as for segmentation of abnormal T2-FLAIR signal across a range of diseases (Duong et al., 2019).

Several prior machine learning approaches have been used to model inter-subject disease heterogeneity, such as distinguishing on an individual subject basis between primary CNS lymphoma and glioblastoma (Wang et al., 2011), or between different types of brain metastases (Kniep et al., 2018). There is evidence that these approaches may be superior to human radiologists (Suh et al., 2018), yet little work has been done to address intra-subject lesion heterogeneity. Notably, one recent study used CNNs to distinguish between WMH due to SVID versus stroke, finding that training a CNN to explicitly distinguish between these diseases allowed for improved correlation between SVID burden and relevant clinical variables (Guerrero et al., 2018). Although a large body of work has detailed methodological approaches to improve segmentation methods for brain tumors, to the best of our knowledge no prior studies have addressed intra-subject disease heterogeneity in the BraTS dataset.

Although the task of distinguishing between different diseases within an individual is typically performed subconsciously by humans, distinguishing between different diseases could be challenging for an automated system if it were not specifically designed and trained to perform such a task. When provided with enough labeled training data, image-based machine learning methods have shown success in identifying patterns that are imperceptible to humans. These include GBM subtypes related to specific genetic mutations (i.e., radiogenomics; Bakas et al., 2017a; Korfiatis et al., 2017; Akbari et al., 2018; Chang et al., 2018; Rathore et al., 2018), or imaging subtypes that are predictive of clinical outcomes (Rathore et al., 2018). Therefore, we sought to train a 3D U-Net model to distinguish between abnormal radiographic signals arising from brain glioma versus WMH in individual subjects, in the mpMRI data of the BraTS 2018 challenge. We hypothesized that this would (1) allow for automatic differentiation of different disease processes, and (2) improve overall accuracy of segmentation of brain tumor extent of disease, particularly in subjects with a large amount of abnormal signal due to WMH.

## Materials and Methods

### Data

We utilized the publicly available data of the BraTS 2018 challenge that describe a multi-institutional collection of pre-operative mpMRI brain scans of 351 subjects (*n*_training_ = 285, and *n*_validation_ = 66) diagnosed with high-grade (glioblastoma) and lower-grade gliomas. The mpMRI scans comprise native T1-weighted (T1), post-contrast T1-weighted (T1PC), T2, and T2-FLAIR scans. Pre-processing of the provided images included re-orientation to LPS (left-posterior-superior) coordinate system, co-registration to the same T1 anatomic template (Rohlfing et al., 2010), resampling to isotropic 1 mm^3^ voxel resolution and skull-stripping as detailed in Bakas et al., 2019. Manual expert segmentation of the BraTS dataset delineated three tumor sub-regions: (1) Necrotic core (NCR), (2) active tumor (enhancing tissue; AT), and (3) peritumoral edematous/infiltrated tissue (ED). The whole tumor extent (WT) was considered the union of all these three classes.

### Manual Annotation of WMH

**FIGURE 1:**
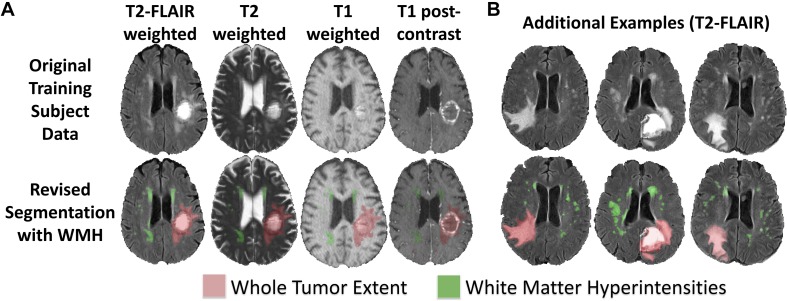
Revised BraTS 2018 training segmentations including annotations of WMH. **(A)** Sample revised segmentation of abnormal signal due to WT (red) and WMH (green) on T2-FLAIR, T2, T1, and T1PC axial slices. **(B)** Three additional example revised segmentation maps for tumor and WMH overlaid on T2-FLAIR axial slices.

In order to define the new tissue class of abnormal signal relating to WMH in the BraTS training subjects, a neuroradiologist (JR; neuroradiology fellow with extensive segmentation experience) defined manually segmentation masks of WMH using ITK-SNAP (Yushkevich et al., 2006). WMH were considered to be abnormal signal due to SVID, chronic infarcts, and/or any periventricular abnormal signal contralateral to the tumor. Examples of these new two class segmentations of the BraTS 2018 dataset are shown in Figure 1.

### U-Net Architecture

We adapted the 3D U-Net architecture (Cicek et al., 2016; Milletari et al., 2016) for voxelwise image segmentation. Our encoder-decoder type fully convolutional deep neural network consists of (1) an encoder limb (with successive blocks of convolution and downsampling encoding progressively deeper/higher-order spatial features), (2) a decoder limb (with a set of blocks – symmetric to those of the encoder limb – of upsampling and convolution, eventually mapping this encoded feature set back onto the input space), and (3) an introduced novel so-called skip connections (whereby outputs of encoding layers are concatenated with inputs to corresponding decoding layers) in order to improve spatial localization over previous generations of fully convolutional networks (3D Res-U-Net; Milletari et al., 2016).

**FIGURE 2:**
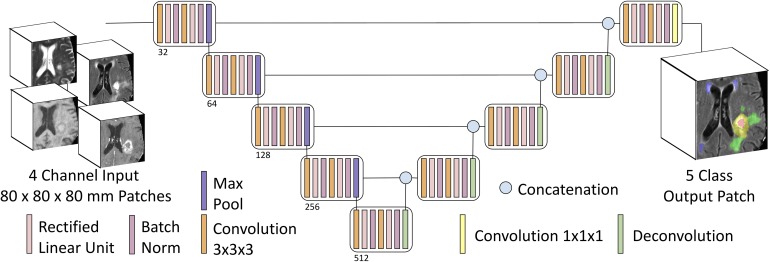
Multiclass input and multiclass output U-Net schematic. Our U-Net has 4-channel input accepting 3D patches from mpMRI with four encoding and decoding layers, and either a four-class output (background, AT, NCR, and ED) or a five-class output (adding WMH as the fifth class).

Our adaptations from the prototypical U-Net architecture included: 4 channel input data (T1, T1PC, T2, T2-FLAIR), 4 or 5 class output data (background = 0, NCR = 1, ED = 2, AT = 4, WMH = 3), with 3D convolutions, and no voxelwise weighting of input label masks. Training patch size was 80 × 80 × 80 voxels (mm), and inference was conducted in the whole image. We zero padded the provided images to increase its size from 240 × 240 × 155 voxels to 240 × 240 × 160 voxels, and hence being divisible by the training input patch size (80 × 80 × 80). Training patch centerpoints were randomly sampled from within the lesion (90%) or from within the whole brain (10%). Train-time data augmentation was performed with random left-right flipping, and constrained affine warps (maximum rotation 45°, maximum scale ±25%, maximum shear ±0.1). Core convolutional blocks included two nodes each of 3D convolution (3 × 3 × 3 kernel, stride = 1, zero padded), rectified linear unit activation, and batch normalization. Four encoding/decoding levels were used, with 32 convolutional filters (channels) in the base/outermost level, and channel number increased by a factor of two at each level (Figure 2).

The network was trained on an NVIDIA Titan Xp GPU (12GB), using the Xavier initialization scheme, Adam optimization algorithm (Kingma and Lei Ba, 2015; initial learning rate 1e^–4^), and 2nd order polynomial learning rate decay over 600 epochs. Training time was approximately 4.5 h. 10-fold internal cross validation on the training set was used for hyperparameter optimization and intrinsic estimation of generalization performance during training. For inference on the validation set, the model was retrained 10 times independently on the *entire* training set (*n* = 285), and model predictions were averaged.

We trained models using this architecture twice; once with the four tissue classes originally annotated in the BraTS dataset, and again with the manual WMH segmentations added as a fifth class. All of the code has been made publicly available at https://github.com/johncolby/svid_paper.

### Performance Metrics

Tissue segmentation performance was evaluated with the Dice metric (2×TP/(2×TP + FP + FN); TP = true positive; FP = false positive; FN = false negative; Dice, 1945) for the tumor segmentation in both models, as well as for WMH in the five-class model. In addition, the 95th percentile of the Hausdorff distance (Hausdorff^95^) was used as a performance evaluation metric, to evaluate the distance between the centers of the predicted and the expert 3D segmentations. The metrics for the four tissue classes and the Hausdorff^95^ distance were measured by submitting our segmentations to the online BraTS evaluation portal^1^ (Davatzikos et al., 2018).

### Further Exploration of U-Net Results

In order to further interrogate the performance of our proposed model, we performed correlations between manually segmented and predicted volumes for WT and WMH, as well as Bland Altman plots to assess agreement between the two measures of tissue volumes for both WT and WMH. For the evaluation of WMH, we performed correlations among the 196 cases that contained at least 100 mm^3^ of WMH. To better understand what could affect performance, we also evaluated correlations between total lesion volumes and Dice scores.

## Results

### Manual WMH Segmentations

Of the manually revised BraTS training data (285 subjects), we found 196 (68.8%) with at least 100 mm^3^ of WMH, 109 (38.4%) with at least 1000 mm^3^ (1 cm^3^) of WMH, 32 (11.2%) with at least 5000 mm^3^ (5 cm^3^) of WMH, and 17 (5.8%) with at least 10000 mm^3^ (10 cm^3^) of WMH. The manual WMH segmentations have been made available for public use at https://github.com/johncolby/svid_paper.

### Segmentation Performance

The performance metrics for the training (10-fold cross validation) and validation subjects (final model) for each of the tissue classes in the four- and five-class models are shown in Table 1. We achieved a median Dice of 0.92 for WT in both the four- and five-class models, in both the training (*p* = 0.52; 10-fold cross validation) and validation datasets (*p* = 0.94). Segmentation performance on AT and tumor core (the union of AT and NCR) were also not significantly different between the four- and five-class models. There were no significant differences between tumor segmentation performance for high- or low-grade gliomas in the training set (*p* = 0.45).

**TABLE 1:** Performance metrics of four- and five-class models applied to the BraTS 2018 Training and Validation datasets.

	Training (*n* = 285)			Validation (*n* = 66)		
Performance metric	4 Class model	5 Class model	*p* value	4 Class model	5 Class Model	*p* value
Dice (Whole Tumor)	0.92 (0.87-0.95)	0.92 (0.87-0.94)	0.52	0.92 (0.89-0.95)	0.92 (0.90-0.95)	0.94
Dice (Enhancing Tumor)	0.82 (0.68-0.88)	0.82 (0.68-0.88)	0.76	0.87 (0.82-0.91)	0.87 (0.81-0.91)	0.99
Dice (Tumor Core)	0.88 (0.75-0.93)	0.89 (0.77-0.93)	0.75	0.91 (0.81-0.95)	0.91 (0.80-0.95)	0.97
Dice (WMH)	N/A	0.42 (0.25-0.55)	N/A	N/A	N/A	N/A
Hausdorff Distance (WT)	3.5 (2.2-9.0)	3.0 (2.2-4.9)	0.002	3.1 (2.0-4.5)	3 (2.0-4.4)	0.84

*Dice scores are median (25th percentile – 75th percentile).*

**FIGURE 3:**
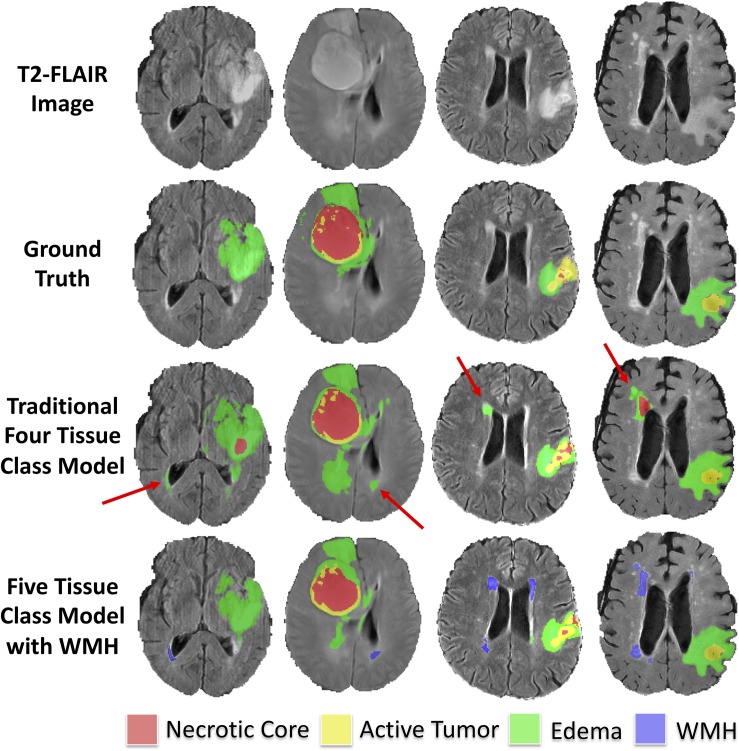
Example segmentations in training subjects with smaller (better) Hausdorff distance metrics in the model with WMH. Axial T2-FLAIR slices of four example training cases are shown in the first row. The ground truth segmentations are overlaid in the second row (background, AT, NCR, and ED). The predicted tumor segmentations overlaid from the four-class model (background, AT, NCR, and ED) and the five-class model (background, AT, NCR, ED, and WMH) are shown in the third and fourth rows, respectively. The red arrows indicate multiple WMH distal to the tumor that were incorrectly classified in the four-class model as either ED or NCR.

**FIGURE 4:**
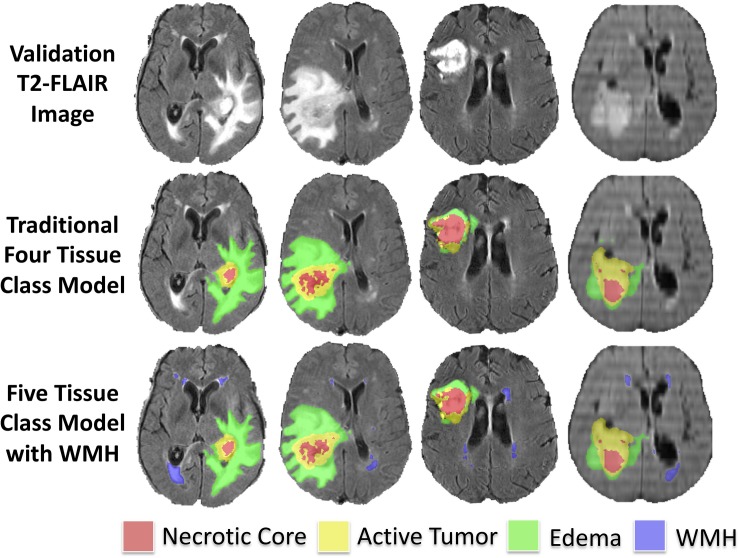
Example predicted segmentations in validation subjects with greater than 5 cm^3^ of WMH. Axial T2-FLAIR slices of four example validation cases are shown in the first row. The predicted tumor segmentations overlaid from the four-class model (background, AT, NCR, and ED) and the five-class model (background, AT, NCR, ED, and WMH) are shown in the second and third row, respectively.

The median Hausdorff^95^ distance in the training data was significantly lower (*p* = 0.002; two tailed *t*-test) in the five-class model (3.0, interquartile range 2.2–9.0) than the traditional four-class model (3.5, interquartile range 2.2–4.9). Example training cases where the Hausdorff^95^ distance were much better in the five-class model are shown in Figure 3 with predicted segmentations for AT, NCR, ED, and WMH for both the four and five-class models. However, the Hausdorff^95^ distance was not significantly different in the validation data (*p* = 0.84). Example validation cases with greater than 5 cm^3^ of WMH are shown in Figure 4, with predicted segmentations for AT, NCR, ED, and WMH.

We achieved a median Dice of 0.42 in the 189 subjects with WMH of at least 100 mm^3^. Median Dice for WMH in subjects with at least 1000 mm^3^ (1 cm^3^), 5000 mm^3^ (5 cm^3^) and 10000 mm^3^ (10 cm^3^) of WMH was 0.52, 0.62, and 0.67, respectively.

### Correlation Between Predicted Lesion Volumes and Manual Segmented Volumes

**FIGURE 5:**
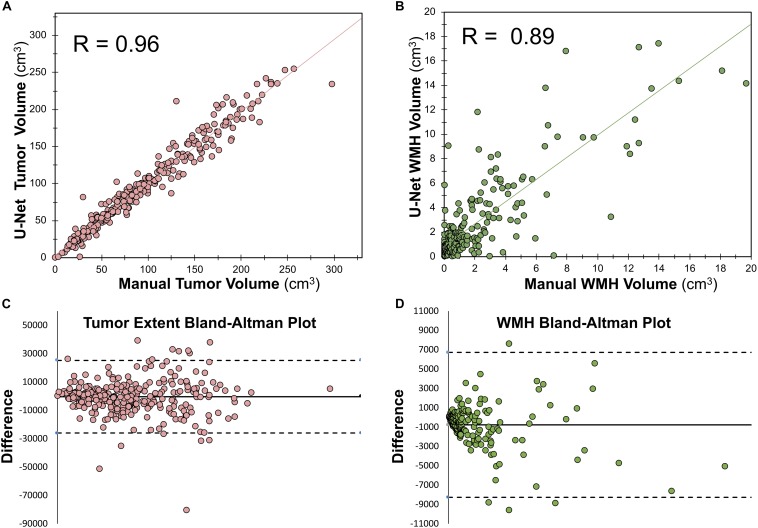
Relationship between manually segmented volume and U-Net predicted volume. **(A)** Pearson correlation between manually segmented tumor volume and U-Net predicted tumor volume. **(B)** Spearman ranked correlation between manually segmented WMH volume and U-Net predicted WMH volume. **(C)** Bland–Altman plot for WT manually segmented volume and U-Net predicted volume. **(D)** Bland–Altman plot for WMH manually segmented volume and U-Net predicted volume. The dotted lines in panels **(C,D)** mark the bounds of the 95% confidence interval of the bias.

Within the training dataset there was a strong correlation between manually segmented WT volume and predicted WT volume (Pearson *r* = 0.96, *p* < 0.0001; Figure 5A). There was also a strong correlation between manually segmented WMH volume and predicted WMH volume (Pearson *r* = 0.89, *p* < 0.0001; Figure 5B). Bland-Altman plots assessing agreement between manual and predicted volume for WT and WMH are shown in Figures 5C,D.

### Correlations Between Lesion Volumes and Dice

**FIGURE 6:**
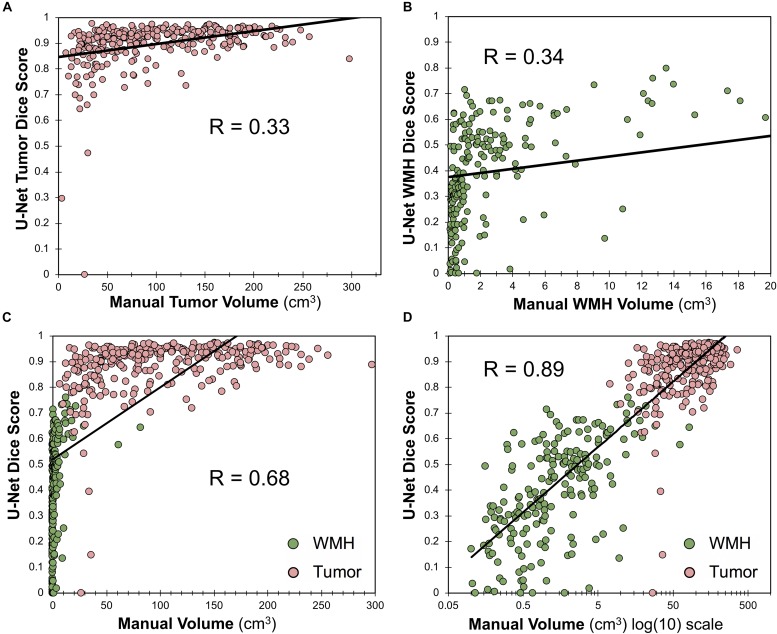
Relationship between Dice score and lesion volume. **(A)** Scatter plot and Pearson correlation between WT volume and WT Dice score. **(B)** Scatter plot and Pearson correlation between WMH volumes and WMH Dice scores. **(C)** Scatter plot and Pearson correlation between volumes and Dice scores for both WMH and WT. **(D)** Scatter plot and Pearson correlation between log(10) transformed volumes and Dice scores for both WMH and WT.

Within the training dataset there was a significant correlation between manually segmented WT volumes and WT Dice scores (Pearson *r* = 0.33, *p* < 0.0001; Figure 6A) and between manually segmented WMH volumes and WMH Dice scores (Pearson *r* = 0.34; *p* < 0.0001; Figure 6B). When combining WT and WMH, there was a stronger correlation between lesion volumes and Dice scores (Pearson *r* = 0.68; *p* < 0.0001; Figure 6C), which was even stronger when the volumes were transformed to a logarithmic (log(10)) scale (Pearson *r* = 0.89; *p* < 0.0001; Figure 6D). There was no significant relationship between WMH volume and WT Dice scores (Pearson *r* = −0.05, *p* = 0.42).

## Discussion

Advanced computational methods are poised to improve diagnostic and treatment methods for patients diagnosed with glioma (Davatzikos et al., 2019; Rudie et al., 2019). However, a critical challenge facing the eventual deployment of artificial intelligence systems into daily clinical practice is disease heterogeneity within subjects. In this study, we utilized the BraTS 2018 dataset and expert-revised WMH segmentations to train a state-of-the-art CNN to successfully distinguish and quantify abnormal signal due to WMH as a distinct tissue class from glioma tissue sub-regions.

We used a 3D CNN (U-Net architecture; Cicek et al., 2016; Milletari et al., 2016) for multiclass tissue segmentation with performance at the top 10% of the BraTS 2018 leaderboard (Bakas et al., 2019; noting that we did not participate in the official competition). U-Nets have been particularly adept at medical image segmentation, due to their ability to convert feature maps obtained during convolutions into a vector and from that vector reconstruct a segmentation, which reduces distortion by preserving the structural integrity.

To our knowledge this is the first study to distinguish intra-subject lesion heterogeneity in the BraTS dataset, noting that Guerrero et al. (2018) previously used a U-Net architecture to distinguish chronic infarcts from WMH due to SVID. Although we hypothesized that adding WMH as a tissue class could improve tumor segmentation performance, we did not find a significant difference between tumor segmentation overlap (Dice) in the model that incorporated WMH as an additional class. Incorporating WMH as a distinct fifth-class did significantly (*p* = 0.002; two tailed *t*-test) improve the Hausdorff (95th percentile) distance metric within the training sample. As the Hausdorff^95^ distance reflects the center of the lesion, and WMH are often far from the tumor, poorer Hausdorff^95^ distance in the four-class model was likely due to false positive segmentations of WMH as tumor as demonstrated in Figure 3. However, upon reviewing validation cases with larger amounts of predicted WMH (Figure 4), it appeared that the original four-class model, although not explicitly trained to model WMH, mostly learned to implicitly ignore most WMH, likely due to spatial characteristics of the WMH being distant from the primary tumors and in characteristic locations and shapes. It is possible that the addition of WMH as an additional class could degrade segmentation performance of ED that was relatively distal to the center of tumor, thus the benefits of reducing distal WMH false positives in the five-class model may have be counterbalanced by increasing false negatives.

As evidenced by the BraTS leaderboard, a Dice of ∼0.90 is considered excellent and has previously been shown to be at a level similar to inter-rater reliability for BraTS (Visser et al., 2019). As demonstrated in Figure 6, we found that lesion volume was an important predictor of Dice scores for both WT (Figure 6A) and WMH (Figure 6B). When evaluating both WT and WMH (Figures 6C,D), we found that the majority of the variance in Dice scores was explained by lesion volume, particularly when transformed to a logarithmic scale (Figure 6D). Thus, poorer performance for WMH in our data appear to largely be driven by smaller lesion sizes. This is consistent with prior literature that has also shown positive correlations between lesion volumes and Dice scores (Winzeck et al., 2018; Duong et al., 2019). Although our reported Dice scores for WMH appear relatively low (0.42), it should be noted that average volume of WMH in the MICCAI 2017 dataset was 16.9 cm^3^ (Kuijf et al., 2019). When looking at cases with larger volumes of WMH (>10 cm^3^) the average Dice score (0.67) was more similar to those reported in the 2017 MICCAI WMH dataset (0.70–0.80; Kuijf et al., 2019). A further explanation for reduced segmentation performance of smaller lesions may be lower inter-rater reliability, such as what has been reported in multiple sclerosis (Dice ∼0.60; Egger et al., 2017). A limitation of the current study is that there is only a single expert annotation for both the BraTS dataset and the WMH, thus the contribution of inter-rater reliability could not be assessed. In the future we also plan to improve detection of smaller lesions by using different neural network architectures, such as two-stage detectors (Girshick et al., 2014), or implementing different loss functions, such a focal loss (Lin et al., 2018; Abraham and Khan, 2019).

As artificial intelligence tools start to become integrated with clinical workflows for more precise quantitative assessments of disease burden, it will be necessary to distinguish, quantify and longitudinally assess a variety of disease processes, in order to assist with more accurate and efficient clinical decision-making. Explicitly tackling intra-subject disease heterogeneity by training models to perform these tasks should help translate these advanced computational methods into clinical practice.

## Data Availability Statement

The datasets generated for this study can be found in the BraTS 2018 dataset https://www.med.upenn.edu/sbia/brats2018/data.html. The manual WMH segmentations and code used in this manuscript have been made available for public use at https://github.com/johncolby/svid_paper.

## Ethics Statement

Ethical review and approval was not required for the study on human participants in accordance with the local legislation and institutional requirements. Written informed consent for participation was not required for this study in accordance with the national legislation and the institutional requirements.

## Author Contributions

JR and JC performed the analysis and prepared the initial manuscript. DW, RS, and JW performed some of the analyses. AR, LS, and SB helped to direct the research. All authors helped to revise the manuscript.

## Conflict of Interest

DW and RS are consultants for the company Galileo CDS and receive consultant fees for their work, which is not directly related to this manuscript. The remaining authors declare that the research was conducted in the absence of any commercial or financial relationships that could be construed as a potential conflict of interest.

## Footnotes

1 https://ipp.cbica.upenn.edu/

## References

[B1] Abraham A. Khan K. (2019). “A novel focal tversky loss function with improved attention u-net for lesion segmentation,” in *Proceedings of the IEEE Comput Soc Conf Comput Vis Pattern Recogn Proceedings - International Symposium on Biomedical Imaging 2019-April*, Piscataway, NJ.

[B2] Akbari H. Bakas S. Pisapia J. M. Nasrallah M. P. Rozycki M. Martinez-Lage M. (2018). In vivo evaluation of EGFRvIII mutation in primary glioblastoma patients via complex multiparametric MRI signature. *Neuro Oncol.* 20 1068–1079. 10.1093/neuonc/noy033 29617843 PMC6280148

[B3] Bakas S. Akbari H. Pisapia J. Martinez-Lage M. Rozycki M. Rathore S. (2017a). *In Vivo* detection of EGFRvIII in glioblastoma via perfusion magnetic resonance imaging signature consistent with deep peritumoral infiltration: the φ-index. *Clin. Cancer Res.* 23 4724–4734. 10.1158/1078-0432.CCR-16-1871 28428190 PMC5559313

[B4] Bakas S. Akbari H. Sotiras A. Bilello M. Rozycki M. Kirby J. S. (2017b). Advancing The cancer genome atlas glioma MRI collections with expert segmentation labels and radiomic features. *Sci. Data* 4:170117. 10.1038/sdata.2017.117 28872634 PMC5685212

[B5] Bakas S. Reyes M. Jakab A. Bauer S. Rempfler M. (2019). Identifying the best machine learning algorithms for brain tumor segmentation, progression assessment, and overall survival prediction in the BRATS challenge. *arXiv* [Preprint]. Available at: https://arxiv.org/abs/1811.02629

[B6] Chang P. Grinband J. Weinberg B. D. Bardis M. Khy M. Cadena G. (2018). Deep-learning convolutional neural networks accurately classify genetic mutations in gliomas. *AJNR Am. J. Neuroradiol.* 39 1201–1207. 10.3174/ajnr.A5667 29748206 PMC6880932

[B7] Chang P. D. (2017). Fully convolutional deep residual neural networks for brain tumor segmentation. *Brain Lesion* 108–118.

[B8] Chartrand G. Cheng P. M. Vorontsov E. Drozdzal M. Turcotte S. Pal C. J. (2017). Deep learning: a primer for radiologists. *Radiographics* 37 2113–2131. 10.1148/rg.2017170077 29131760

[B9] Cicek C. Abdulkadir A. Brox B. Ronneberger R. Lienkamp L. (2016). “3D U-net: learning dense volumetric segmentation from sparse annotation,” in *Medical Image Computing and Computer-Assisted Intervention – MICCAI 2016*, eds Ourselin S. Joskowicz L. Sabuncu M. Unal G. Wells W. (Cham: Springer)

[B10] Clark K. Vendt B. Smith K. Freymann J. Kirby J. Koppel P. (2013). The Cancer Imaging Archive (TCIA): maintaining and operating a public information repository. *J. Digit. Imaging* 26 1045–1057. 10.1007/s10278-013-9622-7 23884657 PMC3824915

[B11] Davatzikos C. Rathore S. Bakas S. Pati S. Bergman M. Kalarot R. (2018). Cancer imaging phenomics toolkit: quantitative imaging analytics for precision diagnostics and predictive modeling of clinical outcome. *J. Med. Imaging* 5:011018. 10.1117/1.JMI.5.1.011018 29340286 PMC5764116

[B12] Davatzikos C. Sotiras A. Fan Y. Habes M. Erus G. Rathore S. (2019). Precision diagnostics based on machine learning-derived imaging signatures. *Magn. Reson. Imaging* 64 49–61. 10.1016/j.mri.2019.04.012 31071473 PMC6832825

[B13] Deng J. Dong W. Socher R. Li L.-J. Li K. Fei-Fei L. (2009). “ImageNet: a large-scale hierarchical image database,” in *Proceedings of the 2009 IEEE Conference on Computer Vision and Pattern Recognition*, Miami, FL.

[B14] Dice L. R. (1945). Measures of the amount of ecologic association between species. *Ecology* 26 297–302. 10.2307/1932409

[B15] Duong M. T. Rudie J. D. Wang J. Xie L. Mohan S. Gee J. C. (2019). Convolutional neural network for automated FLAIR lesion segmentation on clinical brain MR imaging. *AJNR Am. J. Neuroradiol*. 40 1282–1290. 10.3174/ajnr.A6138 31345943 PMC6697209

[B16] Egger C. Opfer R. Wang C. Kepp T. Sormani M. P. Spies L. (2017). MRI FLAIR lesion segmentation in multiple sclerosis: does automated segmentation hold up with manual annotation? *Neuroimage Clin.* 13 264–270. 10.1016/j.nicl.2016.11.020 28018853 PMC5175993

[B17] Fletcher-Heath L. M. Hall L. O. Goldgof D. B. Murtagh F. R. (2001). Automatic segmentation of non-enhancing brain tumors in magnetic resonance images. *Artif. Intell. Med.* 21 43–63. 10.1016/s0933-3657(00)00073-7 11154873

[B18] Girshick G. Donahue D. Darrell D. Malik M. (2014). “Rich feature hierarchies for accurate object detection and semantic segmentation,” in *Proceedings of the IEEE Computer Society Conference on Computer Vision and Pattern Recognition*, Piscataway, NJ.

[B19] Gooya A. Pohl K. M. Bilello M. Cirillo L. Biros G. Melhem E. R. (2012). GLISTR: glioma image segmentation and registration. *IEEE Trans. Med. Imaging* 31 1941–1954. 10.1109/TMI.2012.2210558 22907965 PMC4371551

[B20] Griffanti L. Zamboni G. Khan A. Li L. Bonifacio G. Sundaresan V. (2016). BIANCA (brain intensity AbNormality classification algorithm): a new tool for automated segmentation of white matter hyperintensities. *Neuroimage* 141 191–205. 10.1016/j.neuroimage.2016.07.018 27402600 PMC5035138

[B21] Guerrero R. Qin C. Oktay O. Bowles C. Chen L. Joules R. (2018). White matter hyperintensity and stroke lesion segmentation and differentiation using convolutional neural networks. *Neuroimage Clin.* 17 918–934. 10.1016/j.nicl.2017.12.022 29527496 PMC5842732

[B22] Habes M. Erus G. Toledo J. B. Zhang T. Bryan N. Launer L. J. (2016). White matter hyperintensities and imaging patterns of brain ageing in the general population. *Brain* 139 1164–1179. 10.1093/brain/aww008 26912649 PMC5006227

[B23] Hassabis D. Kumaran D. Summerfield C. Botvinick M. (2017). Neuroscience-inspired artificial intelligence. *Neuron* 95 245–258. 10.1016/j.neuron.2017.06.011 28728020

[B24] Kamnitsas K. Bai W. Ferrante E. McDonagh S. Sinclair M. Pawlowski N. (2017). “Ensembles of multiple models and architectures for robust brain tumour segmentation,” in *Brainlesion: Glioma, Multiple Sclerosis, Stroke and Traumatic Brain Injuries*, eds Crimi A. Bakas S. Kuijf H. Menze B. Reyes M. (Cham: Springer)

[B25] Kingma D. P. Lei Ba J. (2015). “ADAM: a method for stochastic optimization,” in *Proceedings of the Conference Paper at ICLR 2015*, Ithaca, NY.

[B26] Kniep H. C. Madesta F. Schneider T. Hanning U. Schönfeld M. H. Schön G. (2018). Radiomics of brain MRI: utility in prediction of metastatic tumor type. *Radiology* 290 479–487. 10.1148/radiol.2018180946 30526358

[B27] Korfiatis P. Kline T. L. Lachance D. H. Parney I. F. Buckner J. C. Erickson B. J. (2017). Residual deep convolutional neural network predicts MGMT methylation status. *J. Digit. Imaging* 30 622–628. 10.1007/s10278-017-0009-z 28785873 PMC5603430

[B28] Krizhevsky A. Sutskever I. Hinton G. E. (2012). ImageNet classification with deep convolutional neural networks

[B29] Kuijf H. J. Biesbroek J. M. de Bresser J. Heinen R. Andermatt S. Bento M. (2019). Standardized assessment of automatic segmentation of white matter hyperintensities; results of the WMH segmentation challenge. *IEEE Trans. Med. Imaging* 38 2556–2568. 10.1109/TMI.2019.2905770 30908194 PMC7590957

[B30] LeCun Y. Bengio Y. Hinton G. (2015). Deep learning. *Nature* 521 436–444. 10.1038/nature14539 26017442

[B31] Li H. Jiang G. Zhang J. Wang R. Wang Z. Zheng W. (2018). Fully convolutional network ensembles for white matter hyperintensities segmentation in MR images. *ArXiv* [Preprint], 30125711 10.1016/j.neuroimage.2018.07.005

[B32] Lin T. Goyal P. Girshick R. He K. Dollár P. (2018). “Focal loss for dense object detection,” in *Proceedings of the IEEE Transactions on Pattern Analysis and Machine Intelligence*, Piscataway, NJ. 10.1109/TPAMI.2018.2858826 30040631

[B33] Menze B. H. Jakab A. Bauer S. Kalpathy-Cramer J. Farahani K. Kirby J. (2015). The multimodal brain tumor image segmentation benchmark (BRATS). *IEEE Trans. Med. Imaging* 34 1993–2024. 10.1109/TMI.2014.2377694 25494501 PMC4833122

[B34] Milletari F. Navab N. Ahmado S. (2016). “V-net: fully convolutional neural networks for volumetric medical image segmentation,” in *Proceedings of the Fourth International Conference on 3D Vision (3DV)*, Piscataway, NJ.

[B35] Myronenko A. (2019). 3D brain mri tumor segmentation using autoencoder regularization. *Brainles* 11384 311–320. 10.1007/978-3-030-11726-9_28

[B36] Rathore S. Akbari H. Rozycki M. Abdullah K. G. Nasrallah M. P. Binder Z. A. (2018). Radiomic MRI signature reveals three distinct subtypes of glioblastoma with different clinical and molecular characteristics, offering prognostic value beyond IDH1. *Sci. Rep.* 8:5087. 10.1038/s41598-018-22739-2 29572492 PMC5865162

[B37] Rohlfing T. Zahr N. M. Sullivan E. V. Pfefferbaum A. (2010). The SRI24 multichannel atlas of normal adult human brain structure. *Hum. Brain Mapp.* 31 798–819. 10.1002/hbm.20906 20017133 PMC2915788

[B38] Ronneberger O. Fischer P. Brox T. (2015). “U-net: convolutional networks for biomedical image segmentation,” in Medical Image Computing and Computer-Assisted Intervention – MICCAI 2015, eds Navab N. Hornegger J. Wells W. Frangi A. (Cham: Springer), 234–241. 10.1007/978-3-319-24574-4_28

[B39] Rudie J. D. Rauschecker A. M. Bryan R. N. Davatzikos C. Mohan S. (2019). Emerging applications of artificial intelligence in neuro-oncology. *Radiology* 290 607–618. 10.1148/radiol.2018181928 30667332 PMC6389268

[B40] Suh H. B. Choi Y. S. Bae S. Ahn S. S. Chang J. H. Kang S. G. (2018). Primary central nervous system lymphoma and atypical glioblastoma: differentiation using radiomics approach. *Eur. Radiol.* 28 3832–3839. 10.1007/s00330-018-5368-4 29626238

[B41] Visser M. Müller D. M. J. van Duijn R. J. M. Smits M. Verburg N. Hendriks E. J. (2019). Inter-rater agreement in glioma segmentations on longitudinal MRI. *Neuroimage Clin.* 22:101727. 10.1016/j.nicl.2019.101727 30825711 PMC6396436

[B42] Wang S. Kim S. Chawla S. Wolf R. L. Knipp D. E. Vossough A. (2011). Differentiation between glioblastomas, solitary brain metastases, and primary cerebral lymphomas using diffusion tensor and dynamic susceptibility contrast-enhanced MR imaging. *AJNR Am. J. Neuroradiol.* 32 507–514. 10.3174/ajnr.A2333 21330399 PMC8013110

[B43] Wardlaw J. M. Valdés Hernández M. C. Muñoz-Maniega S. (2015). What are white matter hyperintensities made of? Relevance to vascular cognitive impairment. *J. Am. Heart Assoc.* 4:001140. 10.1161/JAHA.114.001140 26104658 PMC4599520

[B44] Winzeck S. Hakim A. McKinley R. Pinto J. A. Alves V. Silva C. (2018). ISLES 2016 and 2017-benchmarking ischemic stroke lesion outcome prediction based on multispectral MRI. *Front. Neurol.* 9:679. 10.3389/fneur.2018.00679 30271370 PMC6146088

[B45] Yushkevich P. A. Piven J. Hazlett H. C. Smith R. G. Ho S. Gee J. C. (2006). User-guided 3D active contour segmentation of anatomical structures: significantly improved efficiency and reliability. *Neuroimage* 31 1116–1128. 10.1016/j.neuroimage.2006.01.015 16545965

